# An artificial lncRNA targeting multiple miRNAs overcomes sorafenib resistance in hepatocellular carcinoma cells

**DOI:** 10.18632/oncotarget.12304

**Published:** 2016-09-28

**Authors:** Shuyao Tang, Gang Tan, Xian Jiang, Peng Han, Bo Zhai, Xuesong Dong, Haiquan Qiao, Hongchi Jiang, Xueying Sun

**Affiliations:** ^1^ The Hepatosplenic Surgery Center, Department of General Surgery, the First Affiliated Hospital of Harbin Medical University, Harbin, China; ^2^ Department of General Surgery, the Fourth Affiliated Hospital of Harbin Medical University, Harbin, China

**Keywords:** sorafenib, hepatocellular carcinoma, drug resistance, LncRNA, miRNA

## Abstract

Sorafenib resistance remains a major obstacle for the effective treatment of hepatocellular carcinoma (HCC), and a number of miRNAs contribute to this resistance. However, the regulatory networks of miRNAs are very complex, thus inhibiting a single miRNA may sequentially activate other compensatory pathways. In the present study, we generated an artificial long non-coding RNA (AlncRNA), which simultaneously targets multiple miRNAs including miR-21, miR-153, miR-216a, miR-217, miR-494 and miR-10a-5p. These miRNAs have been shown to be upregulated in sorafenib-resistant cells and participate in the mechanisms underlying sorafenib resistance. The AlncRNA contains tandem sequences of 6 copies of the complementary binding sequences to the target miRNAs and is expressed by an adenoviral vector (Ad5-AlncRNA). Infection of Ad5-AlncRNA into sorafenib-resistant HCC cells blocked the function of miRNAs, and sequentially inhibited the downregulation of PTEN and activation of AKT. Ad5-AlncRNA significantly inhibited proliferation and induced apoptosis of sorafenib-resistant cells and enhanced the effects of sorafenib *in vitro* and in animal models. Inhibition of autophagy decreased the sensitivity of sorafenib-resistant cells to Ad5-AlncRNA, while its induction had the opposite effect. These results indicate that targeting multiple miRNAs by the artificial lncRNA could be a potential promising strategy for overcoming sorafenib resistance in the treatment of HCC.

## INTRODUCTION

Hepatocellular carcinoma (HCC) is the second leading cause of cancer-related death in men worldwide, and is extremely resistant to chemotherapy [1]. Sorafenib is a first-line chemotherapeutic agent for treating advanced HCC, but has only limited survival benefits [2, 3]. It remains the only systemic drug as no alternative effective agents have proven superior to sorafenib in treating HCC [4]. However, some HCC patients initially respond to sorafenib but eventually succumb to the disease, indicating acquired resistance to the drug [5]. Therefore, it is important to unravel the molecular mechanisms that underlie sorafenib resistance in HCC cells and develop potential strategies capable of overcoming such resistance.

MicroRNAs (miRNAs) play critical roles in regulating multiple cellular functions by post-transcriptional gene silencing [6]. A number of miRNAs have been shown to contribute to the progression of HCC [7]. Among these, miRNA-21 is one of the well characterized miRNAs involved in various types of malignancies, including HCC, by suppressing several tumor suppressor genes [8, 9]. MiR-21 is also a therapeutic target for reversing drug resistance in various human cancers [10]. We have recently reported that miR-21 contributes to sorafenib resistance of HCC cells by inhibiting autophagy via the phosphatase and tensin homolog (PTEN)/AKT pathway [11]. A few other miRNAs have also been shown to participate in the mechanisms underlying sorafenib resistance. Overexpression of miR-153 counteracts the inhibitory effect of sorafenib against HCC cells [12]. MiR-216a/217 activates the PI3K/AKT and TGF-β pathways, contributing to sorafenib resistance in HCC cells [13]. MiR-222 enhances the resistance of HCC cells to sorafenib through activating the AKT signaling pathway [14]. MiR-494 increases sorafenib resistance in HCC cells by targeting PTEN [15]. These results indicate that these miRNAs may represent potential therapeutic targets for overcoming sorafenib resistance in HCC cells.

However, each of the above studies has investigated the role of a single miRNA or an miRNA cluster. The regulatory networks of miRNAs are extremely complex; one miRNA regulates multiple target genes, while one target gene is regulated by multiple miRNAs [6]. Inhibiting one miRNA has a limited effect as it may simultaneously or sequentially activate other compensatory or bypassing pathways. Therefore, we hypothesize that simultaneously targeting multiple miRNAs with complementary functions that contribute to the same event may achieve greater efficacy in reversing sorafenib resistance in HCC cells.

Recently, long non-coding RNAs (lncRNAs) have emerged as important functional transcripts, and become attractive potential therapeutic targets for various types of cancers, including HCC [16, 17]. These natural lncRNAs serve as tumor oncogenic suppressors or promoters through a variety of chromatin-based mechanisms or via cross-talk with other RNA species [16]. LncRNAs have recently been discovered to act as competing endogenous RNA (ceRNA) and function as “molecular sponges” for miRNAs [18]. On the other hand, miRNAs bind to mRNAs of target genes to induce mRNA degradation or inhibit mRNA translation, thereby exerting their role as post-transcriptional regulators [6]. Based on the functions of miRNAs and lncRNAs, we designed an artificial lncRNA (AlncRNA), which contains tandem antisense sequences that specifically bind the six miRNAs involved in sorafenib resistance. We investigated whether transfection of the AlncRNA by an adenoviral vector could simultaneously block the function of multiple miRNAs, thereby reversing sorafenib resistance in HCC cells.

## RESULTS

### Downregulation of PTEN and activation of AKT in sorafenib-resistant HCC cells

Two sorafenib-resistant cell lines, termed HepG2-SR and Huh7-SR, were generated from HCC cell lines, HepG2 and Huh7, respectively, as described in MATERIALS AND METHODS. Sorafenib showed a significantly weaker inhibitory effect on the proliferation of sorafenib-resistant cells than their respective parental cells in a dose-dependent manner (Supplementary Figure S1A). The values of IC_50_ were 5.76 and 20.13 μM for HepG2 and HepG2-SR, respectively; and they were 7.38 and 27.45 μM for Huh7 and Huh7-SR cells, respectively. When the concentrations of sorafenib reached above 20 μM, the growth of parental cells were almost completely arrested by sorafenib, while the viability of HepG2-SR or Huh7-SR continued to remain around 35–60% (Supplementary Figure S1A). These results are in agreement with apoptosis results, thus sorafenib-resistant cells showed resistant to sorafenib-induced apoptosis (Supplementary Figure S1B). Compared with parental cells, sorafenib-resistant cells expressed lower levels of PTEN and higher levels of p-AKT; while the expression of AKT remained similar between sorafenib-resistant and parental cells (Supplementary Figure S1C). Incubation of sorafenib decreased the level of PTEN and increased the level of p-AKT in both parental and sorafenib-resistant cells; and sorafenib-resistant cells expressed lower levels of PTEN and higher levels of p-AKT than the respective parental cells, following similar incubation (Supplementary Figure S1C). On the other hand, sorafenib induced stronger activation of caspase-3 in parental cells than in sorafenib-resistant cells (Supplementary Figure S1C). These results indicate that the PTEN/AKT pathway is involved in sorafenib resistance in HCC cells, supporting previous reports [8, 11, 12, 14, 15, 19].

### Selection of miRNA targets and construction of AlncRNA

**Figure 1:**
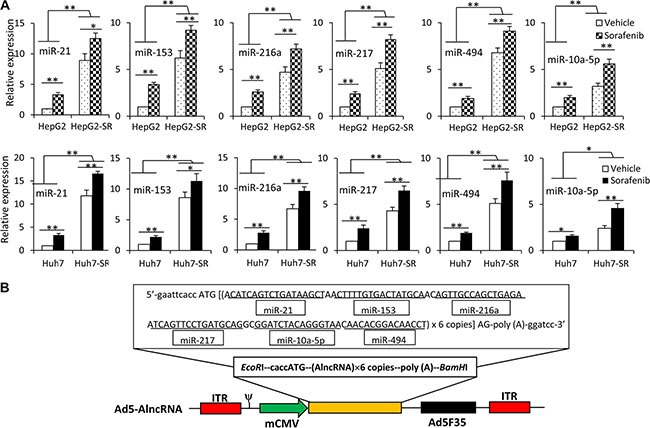
Exposure to sorafenib induces upregulation of multiple miRNAs (**A**) HepG2, HepG2-SR, Huh7 and Huh7-SR cells were incubated with sorafenib (2.5 μM) for 96 h, and then the expression of miRNAs was measured by real-time RT-PCR. The level of miRNA from untreated parental cells was defined as 1. “*” (*P* < 0.05) and “**” (*P* < 0.001) indicate a significant difference. (**B**) Schematic diagram of Ad5-AlncRNA. The expression cassette of whole-length encoding sequence of AlncRNA was inserted into the *EcoR*I/*BamH*I sites of adenovirus E1 region, to generate the recombinant adenovirus Ad5-AlncRNA. ITR, inverted terminal repeats; ψ, adenovirus 5 packaging signal; mCMV, mouse cytomegalovirus promoter.

We analyzed the miRNA expression profiles in Huh7 and Huh7-SR cells by using a Human MicroRNA Array. Pairwise significance analysis of the data indicated that the expression of 18 miRNAs (Let-7b, Let-7c, miR-10a-5p, miR-10b-5p, miR-34-a, miR-21, miR-30a-3p, miR-195, miR-216a, miR-217, miR-219-1-3p, miR-223, miR-494, miR-616, miR-664, miR-1260, miR-1274a, miR-1291) was significantly higher in Huh7-SR cells, compared with Huh7 cells. We also performed a systematic review of English articles by searching PubMed, EMBASE, Web of Science and Google scholar, and found that 6 miRNAs (miR-21, miR-153, miR-216a, miR-217, miR-222, miR-494) have been reported to contribute to sorafenib resistance in HCC [8, 11–15, 19]. After deducting the duplicates, a total of 20 miRNA candidates (Let-7b, Let-7c, miR-10a-5p, miR-10b-5p, miR-34-a, miR-21, miR-30a-3p, miR-153, miR-195, miR-216a, miR-217, miR-219-1-3p, miR-222, miR-223, miR-494, miR-616, miR-664, miR-1260, miR-1274a, miR-1291) were identified. To validate the expression patterns of these 20 miRNAs, real-time RT-PCR was used to measure their expression. Six miRNAs (miR-10a-5p, miR-21, miR-153, miR-216a, miR-217, miR-494), with the most elevated expression in sorafenib-resistant cells compared with the respective parental cells, were selected as targets (Supplementary Table S1). We further examined the expression of the above 6 miRNAs in HCC cells by exposing them to sorafenib (2.5 μM) for 96 h. Incubation of either parental or sorafenib-resistant HCC cells with sorafenib upregulated the expression of these 6 miRNAs, although sorafenib-resistant cells expressed higher levels of miRNAs than their parental cells, in the presence or absence of sorafenib (Figure 1A). These results confirmed further that the above 6 miRNAs were involved in sorafenib resistance. Therefore, we selected them as targets and designed an AlncRNA, which contained tandem antisense sequences (6 copies) specifically binding the 6 miRNAs (Supplementary Table S2). The sequence of the AlncRNA was inserted into an adenoviral vector to construct Ad5-AlncRNA (Figure 1B).

### Efficiency of adenovirus infection and AlncRNA expression

**Figure 2:**
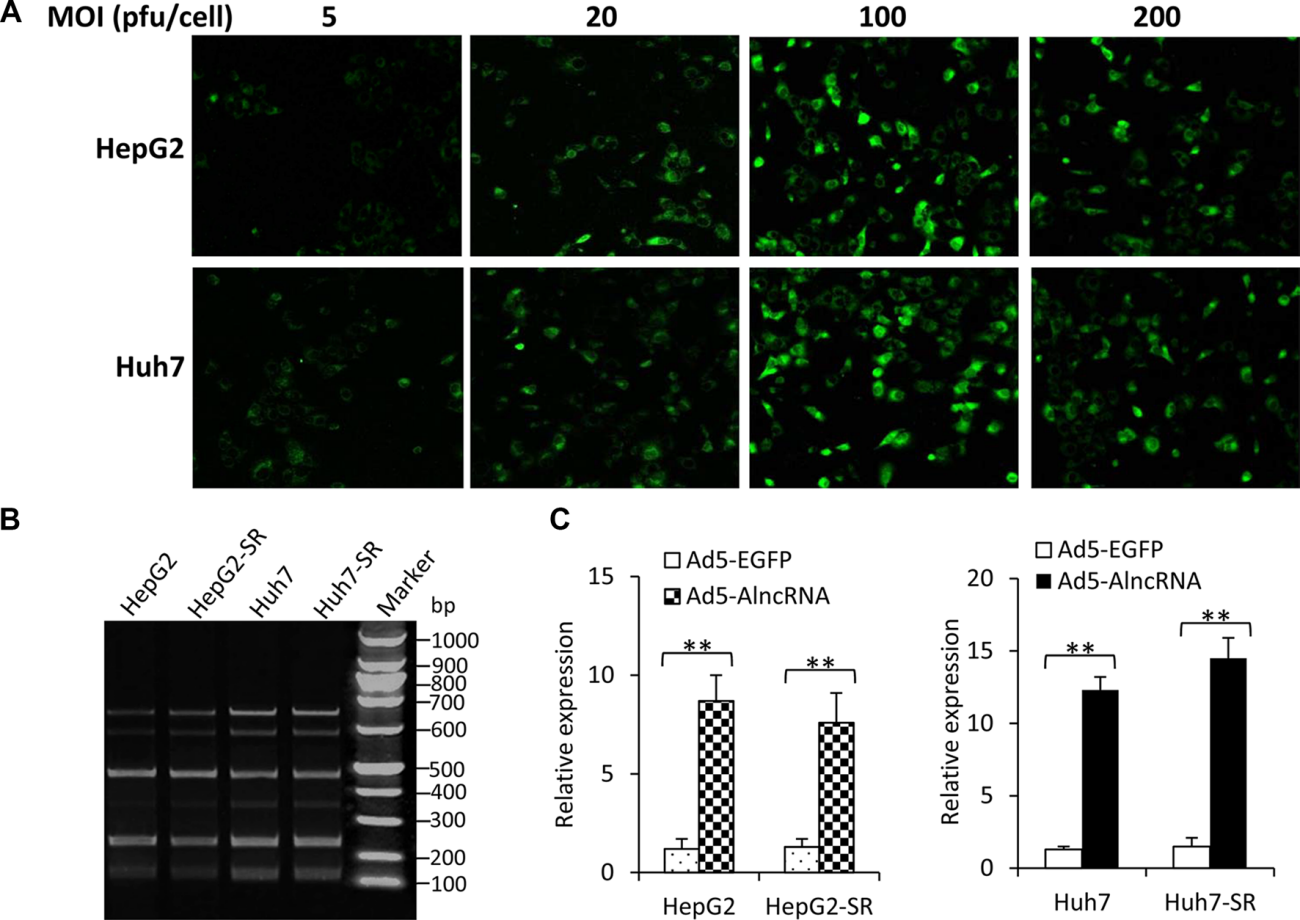
Expression of AlncRNA in HCC cells after Ad5-AlncRNA infection (**A**) HepG2 and Huh7 cells were infected with Ad5-EGFP at MOIs of 10, 20, 100 and 200 pfu/cell for 3 h, and then observed under fluorescent microscopy. (**B**, **C**) HepG2, HepG2-SR, Huh7 and Huh7-SR cells were infected with Ad5-AlncRNA at an MOI of 100 pfu/cell for 3 h. Cells were harvested and subjected to conventional RT-PCR and PCR products were electrophoresed (B), or real-time PCR to quantify the expression of AlncRNA (C). “**” (*P* < 0.001) indicates a significant difference.

The infection efficiency of Ad5-AlncRNA was first examined by incubating HepG2 and Huh7 cells with Ad5-AlncRNA at MOIs of 10, 20, 100 and 200 pfu/cell. Ad5-EGFP served as an internal control and EGFP expression was detected by observing cells under fluorescent microscopy. The infection efficiency reached a peak when the MOI was at 100 pfu/cell. The cells infected with adenoviruses at MOI of 200 pfu/cell contained fewer EGFP-expressing cells than those at MOI of 100 pfu/cell, indicating that an MOI of 100 pfu/cell might be an optimal concentration and was used in the following experiments (Figure 2A). HepG2, HepG2-SR, Huh7 and Huh7-SR cells were infected with Ad5-AlncRNA at an MOI of 100 pfu/cell for 3 h. Cells were harvested and subjected to RT-PCR assays with a pair of primers. The AlncRNA contained the complementary sequences of 6 target miRNAs, which were repeated 6 times. Thus, PCR amplification resulted in 6 fragments of PCR products with different molecular sizes (Figure 2B). Quantitative RT-PCR assays further confirmed that infection of Ad5-AlncRNA, but not Ad5-EGFP, led to the expression of AlncRNA genes in the above cells (Figure 2C).

### Infection of AlncRNA increases PTEN expression and inhibits AKT activation

**Figure 3:**
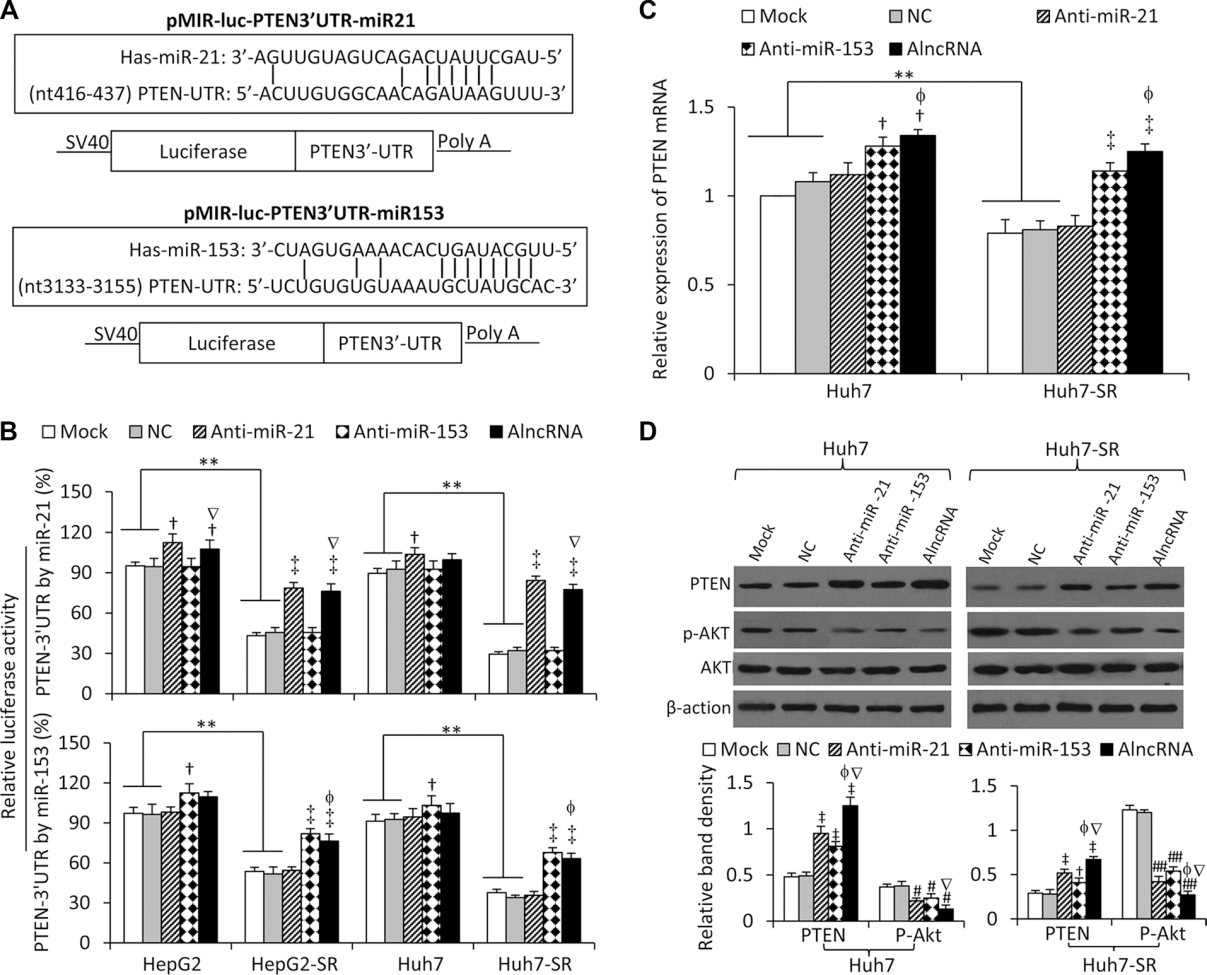
AlncRNA increases PTEN expression and inhibits AKT phosphorylation by targeting miR-21 and miR-153 (**A**) Predicted paring of miR-21 (or miR-153) to the 3′-UTR of the human PTEN gene, and the diagram of a pMIR-REPORT luciferase reporter vector containing the 3′-UTR of PTEN with miR-21 (or miR-153) seed site (pMIR-luc-PTEN3′UTR-miR21 or pMIR-luc-PTEN3′UTR-miR153). (**B**) Luciferase activities in HepG2, HepG2-SR, Huh7 and Huh7-SR cells transfected with pMIR-luc-PTEN3′UTR-miR21, pMIR-luc-PTEN3′UTR-miR153 or a control vector. Cells were mock co-transfected or co-transfected with negative control (NC), anti-miR-21 or anti-miR153 oligonucleotides, or infected with AlncRNA. Relative luciferase activity was calculated as the percentage of luciferase activity in luciferase reporter vectors -transfected cells over those with the control vector. (**C**, **D**) Huh7 and Huh7-SR cells were mock transfected or transfected with NC, anti-miR-21, anti-miR153 or AlncRNA. Twenty-four hours later, cells were subjected to real-time RT-PCR to measure PTEN mRNA expression (C), or immunoblotted for detecting protein expression (D). The density of each band was measured and normalized to respective β-actin. “**” (*P* < 0.001) indicates a significant difference. “†” (*P* < 0.05) and “‡” (*P* < 0.001) indicate a significant increase; while “#” (*P* < 0.05) and “##” (*P* < 0.001), a significant reduction, versus mock transfected cells. “f” (*P* < 0.05) indicates a significant difference from anti-miR-21-transfected cells, while “Ñ” (*P* < 0.05), from anti-miR-153-transfected cells.

We next examined whether Ad5-AlncRNA infection could alter the expression of the targeted miRNAs by using quantitative RT-PCR. Again, sorafenib-resistant cells were shown to express higher levels of the 6 miRNAs than their respective parental cells, but infection of Ad5-AlncRNA had little effect on the expression of the miRNAs (Supplementary Figure S2). PTEN has been reported to be a common target of the 6 miRNAs aforementioned [8, 11–13, 15, 20]. We, therefore, investigated whether Ad5-AlncRNA could regulate the expression of PTEN by inhibiting the function of miRNAs, and miR-21 and miR-153 were selected as the example target miRNAs. Two luciferase reporters containing the 3′-UTR of PTEN with a miR-21 or miR-153 seed site were generated (Figure 3A). In agreement with the upregulation of miR-21, the luciferase activities of PTEN-3′UTR by miR-21 were suppressed by 54.3 ± 3.1% and 68.3 ± 4.2% in HepG2-SR and Huh7-SR cells, compared with their respective parental cells; and co-transfection of anti-miR-21 or AlncRNA, but not anti-miR-153 significantly attenuated this suppression by miR-21 (Figure 3B). On the other hand, the luciferase activities of PTEN-3′UTR by miR-153 were suppressed by 43.9 ± 5.6% and 53.7 ± 5.1% in HepG2-SR and Huh7-SR cells, compared with their respective parental cells, and co-transfection of anti-miR-153 or AlncRNA, but not anti-miR-21, significantly attenuated this suppression (Figure 3B). The results indicate that AlncRNA could inhibit the function of miR-21 and miR153 in regulating PTEN.

The above results were supported by the expression of PTEN. Huh7-SR cells expressed lower levels of PTEN mRNA than Huh7 cells (Figure 3C), in agreement with PTEN protein expression (Supplementary Figure S1C). Transfection of anti-miR-21 had little effect on the expression of PTEN mRNA, while anti-miR-153 or AlncRNA significantly increased PTEN mRNA expression (Figure 3C). Both anti-miR-21 and anti-miR-153 significantly increased the expression of PTEN protein, and AlncRNA showed a stronger effect than either anti-miR-21 or anti-miR-153 (Figure 3D). These results indicate that miR-21 may prevent PTEN mRNA from being translated only, while miR-153 could induce PTEN mRNA degradation. In line with the PTEN expression data, the phosphorylation of AKT was significantly reduced by anti-miR-21 and miR-153, while AlncRNA showed a stronger effect than either anti-miR-21 or miR-153 (Figure 3D).

### AlncRNA enhances the inhibitory effects of sorafenib on sorafenib-resistant HCC cells by targeting the PTEN/AKT pathway

**Figure 4:**
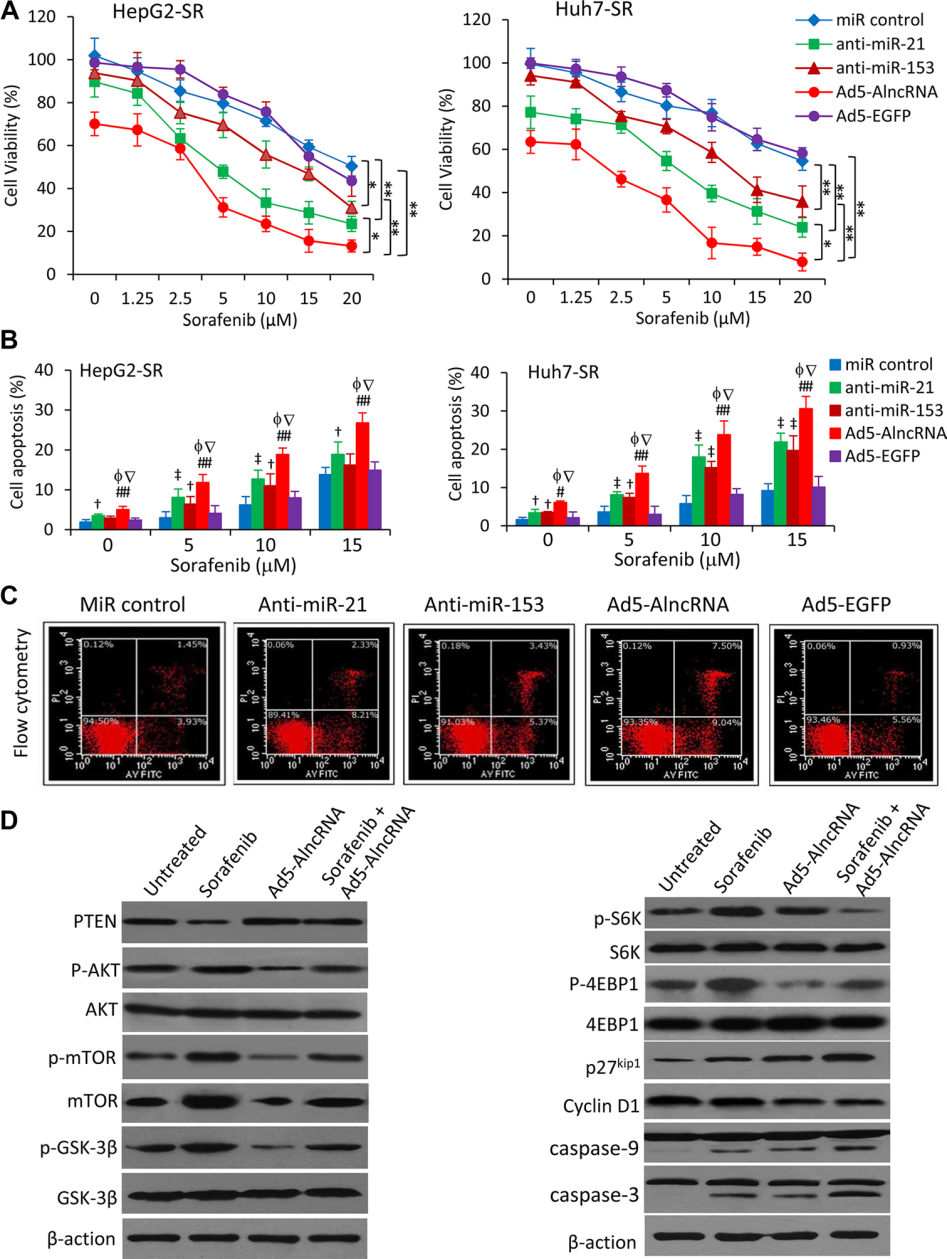
AlncRNA enhances the effects of sorafenib-induced growth inhibition and apoptosis in sorafenib-resistant HCC cells (**A**) HepG2-SR or Huh7-SR cells transfected with miRNA negative control, anti-miR-21 or anti-miR-153 oligonucleotides, or infected with Ad5-AlncRNA or Ad5-EGFP, were incubated with serial concentrations of sorafenib for 48 h. Cell viability (%) was measured and normalized with the corresponding untreated cells. (**B**) The above cells incubated with sorafenib (0, 2.5, 5 or 10 μM) for 48 h were analyzed to measure apoptosis rates (%). (**C**) Representative dot plots were from the above cytometrically analyzed Huh7-SR cells incubated with sorafenib (5 μM). (**D**) Huh7-SR cells untreated or incubated with sorafenib (5 μM), or infected with Ad5-AlncRNA, or the combination for 48 h. Cells were then subjected to immunoblotting. “*” (*P* < 0.05) and “**” (*P* < 0.001) indicate a significant difference. “†” (*P* < 0.05)and “‡” (*P* < 0.001) indicate a significant increase from miR control-treated cells. “#” (*P* < 0.05) and “##” (*P* < 0.001) indicate a significant increase from Ad5-EGFP-infected cells. “f” (*P* < 0.05) indicates a significant difference from anti-miR-21-transfected cells, while “Ñ” (*P* < 0.05), from anti-miR-153-transfected cells.

The above results indicate that AlncRNA exerts a stronger ability in regulating the PTEN/AKT pathway in HCC cells. Thus, we first examined the effect of Ad5-AlncRNA alone on cell proliferation. Infection of Ad5-AlncRNA showed a stronger inhibitory effect on sorafenib-resistant HCC cells compared with the respective parental cells in a dose- (Supplementary Figure S3A) and time- (Supplementary Figure S3B) dependent manner. We then examined whether Ad5-AlncRNA could enhance the effects of sorafenib on the viability and apoptosis of sorafenib-resistant HCC cells, with anti-miR-21 and anti-miR-153 as positive controls [8, 11, 12]. Transfection of both anti-miR-21 and anti-miR-153 oligonucleotides enhanced the inhibitory effects of sorafenib in sorafenib-resistant HCC cells (Figure 4A). Infection of Ad5-AlncRNA showed an even stronger inhibitory effect of sorafenib, than either anti-miR-21 or anti-miR-153, in sorafenib-resistant cells (Figure 4B). These results were supported by apoptosis assays, which showed AlncRNA displayed stronger effects than either anti-miR-21 or anti-miR-153, in enhancing the pro-apoptotic activity of sorafenib in sorafenib-resistant cells (Figure 4B, 4C).

Sorafenib induced the downregulation of PTEN, increased the expression of p-AKT and mTOR, and increased the phosphorylation of mTOR, GSK3β, S6K and 4EBP1 in Huh7-SR cells (Figure 4D). Sorafenib also induced the downregulation of p27 and upregulation of cyclin D1 (Figure 4D). Ad5-AlncRNA infection increased the expression of PTEN and decreased the expression of p-AKT. Upon the infection of Ad5-AlncRNA, the phosphorylation of mTOR, GSK3β, S6K and 4EBP1 was reduced, sequentially p27 expression was increased while cyclin D1 expression was reduced (Figure 4D). Both sorafenib and Ad5-AlncRNA increased, and their combination further increased the activation of caspase-9 and caspase-3 (Figure 4D).

### Increased autophagy by AlncRNA contributes to its effect on sorafenib-resistant HCC cells

**Figure 5:**
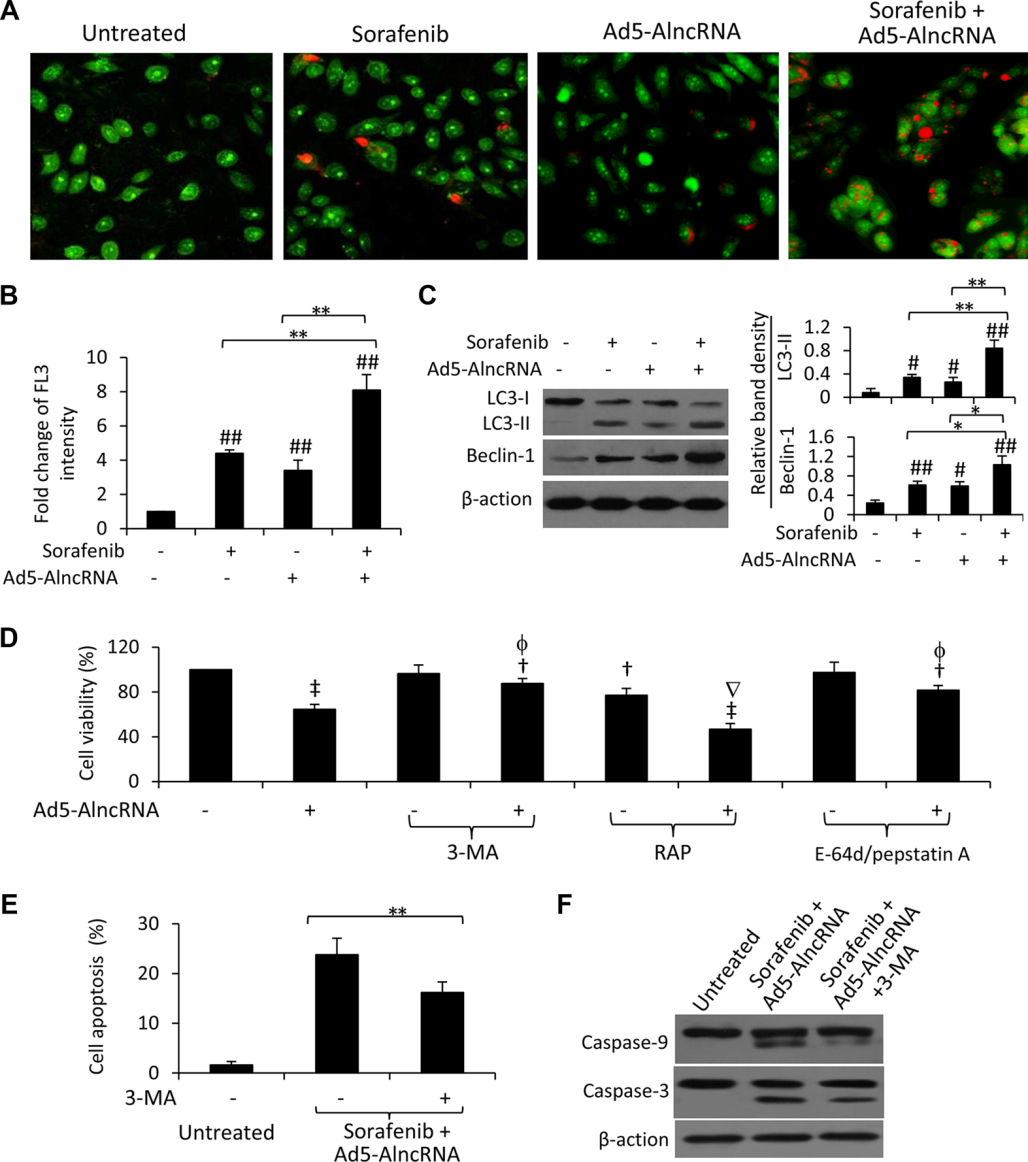
AlncRNA enhances sorafenib-induced autophagy in sorafenib-resistant cells Untreated or Ad5-AlncRNA-infected Huh7-SR cells were incubated in the presence or absence of sorafenib (5 μM) for 48 h. (**A**) Representative images were taken from acridine orange-stained cells. (**B**) The above cells were subjected to flow cytometry, and the fold change of acridine orange fluorescence intensity (FL3) versus untreated cells was calculated. (**C**) The above cells were immunoblotted. The density of each band was measured and normalized to respective β-actin. (**D**) Untreated or Ad5-AlncRNA-infected cells were incubated for 48 h in the presence or absence of 3-MA (3-methyladenine) (5 mM), RAP (rapamycin) (10 nM), or a mixture of E-64d (10 μg/ml) and pepstatin A (10 μg/ml). Cell viability (%) was compared to untreated cells. (**E**, **F**) Huh7-SR cells infected with Ad5-AlncRNA were incubated with sorafenib (5 μM) for 48 h in the presence or absence of 3-MA (5 mM). Cells was subjected to apoptosis assays (E) and immunoblotting (F). Untreated cells served as controls. “*” (*P* < 0.05) and “**” (*P* < 0.001) indicate a significant difference. “#” (*P* < 0.05) and “##” (*P* < 0.001) indicate a significant increase, while “†” (*P* < 0.05) and “‡” (*P* < 0.001), a significant reduction, from untreated control cells. “f” (*P* < 0.05) indicates a significant difference, while “Ñ” (*P* < 0.05), a significant reduction, from Ad5-AlncRNA-infected cells.

We have previously demonstrated that autophagy participates in the mechanisms of sorafenib resistance in HCC cells [21], and inhibition of miR-21 enhances the efficacy of sorafenib against sorafenib-resistant HCC cells by enhancing autophagic cell death [11]. Here, we showed that Ad5-AlncRNA and sorafenib significantly increased autophagy of Huh7-SR cells since cells had more acridine orange-stained AVOs than untreated cells; while their combination induced even more numerous autophagic cells (Figure 5A). These results are in agreement with quantitative analysis by flow cytometry (Figure 5B). Immunoblotting analysis of the expression of key autophagic proteins showed that Ad5-AlncRNA and sorafenib both increased, and in combination further increased the expression of LC3-II and Beclin-1, in Huh7-SR cells (Figure 5C).

Suppression of autophagy by 3-MA inhibited AlncRNA-induced reduction in the viability of Huh7-SR cells. In contrast, RAP, an inducer of autophagy [22], increased AlncRNA- and sorafenib-induced growth inhibition (Figure 5D). To further verify the role of autophagy in AlncRNA-induced reduction in cell viability, we applied a mixture of E-64d (10 μg/ml) and pepstatin A (10 μg/ml), which are lysosomal protease inhibitors that suppresses the formation of autophagolysosomes and late-stage autophagy. E-64d/pepstatin A attenuated Ad5-AlncRNA-induced reduction in the viability of sorafenib-resistant cells (Figure 5D). In addition, inhibition of autophagy by 3-MA counteracted the pro-apoptotic effect (Figure 5E) and the activation of caspase-9 and −3 (Figure 5F) by the combination of sorafenib and Ad5-AlncRNA.

### AlncRNA enhances the efficacy of sorafenib in treating sorafenib-resistant HCC tumors *in vivo*

**Figure 6:**
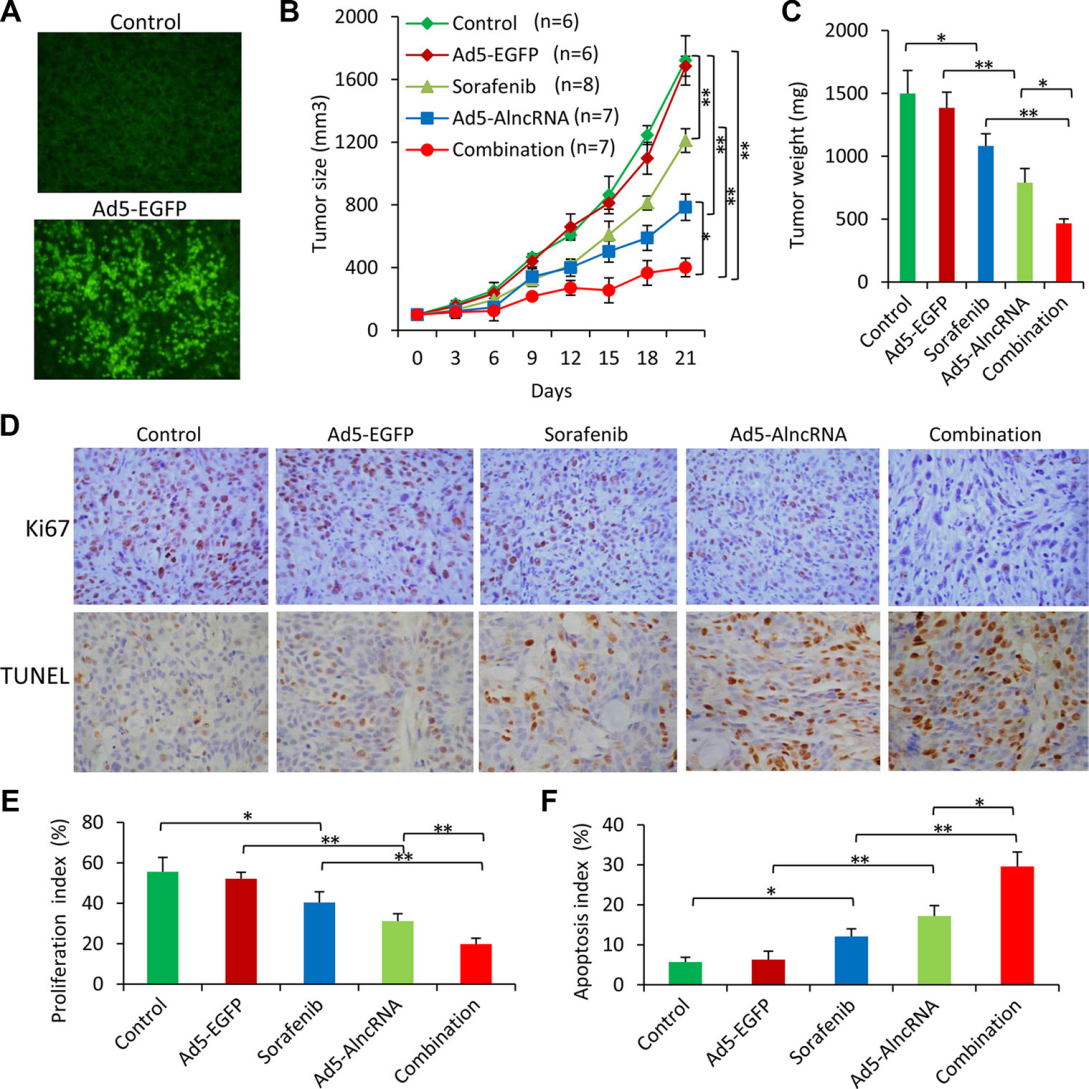
AlncRNA enhances the efficacy of sorafenib to suppress sorafenib-resistant tumors *in vivo* Subcutaneous tumors were established in mice, which received different treatments for 21 days as described in MATERIALS AND METHODS. (**A**) Illustrated are representative fluorescence microscopy-viewed tumor sections from vehicle and Ad5-EGFP-treated mice prepared 10 days after commencement of treatments. (**B**) The sizes of tumors were recorded. (**C**) Tumors were excised and weighed at the end of experiments. (**D**) Sections of tumors were stained with an anti-Ki67 Ab (upper panel, magnification ×100) or TUNEL (middle panel, magnification ×200). (**E, F**) Proliferation index (E) and apoptosis index (F) were quantified. “*” (*P* < 0.05) and “**” (*P* < 0.001) indicate a significant difference.

Tumors injected with Ad5-EGFP showed an intense expression of EGFP (Figure 6A), but had little effect on the growth of tumors (Figure 6B, 6C), compared with control tumors. Oral administration of sorafenib and intratumoral injection of Ad5-AlncRNA significantly inhibited tumor growth (Figure 6B), and reduced the weight of tumors by 27.7% and 43.6%, compared with control tumors, respectively, at day 21 (Figure 6C). The combination therapy resulted in a further reduction of tumor size and weight, compared with sorafenib or Ad5-AlncRBA alone (Figure 6B, 6C).

There were fewer Ki-67 positive cells in tumors treated with sorafenib or Ad5-AlncRNA, compared with control tumors, and the combination therapy resulted in even fewer Ki-67 positive cells (Figure 6D, 6E). Tumors treated with sorafenib or Ad5-AlncRNA had a greater number of TUNEL-positive cells than control tumors, respectively, and the combination therapy resulted in even more TUNEL-positive cells (Figure 6D, 6F).

The expression of key genes regulated by miRNAs involved in this study was examined by immunohistochemistry (Supplementary Figure S4A) and immunoblotting analysis (Supplementary Figure S4B); and the results were consistent with *in vitro* experiments (Figure 4D).

## DISCUSSION

**Figure 7:**
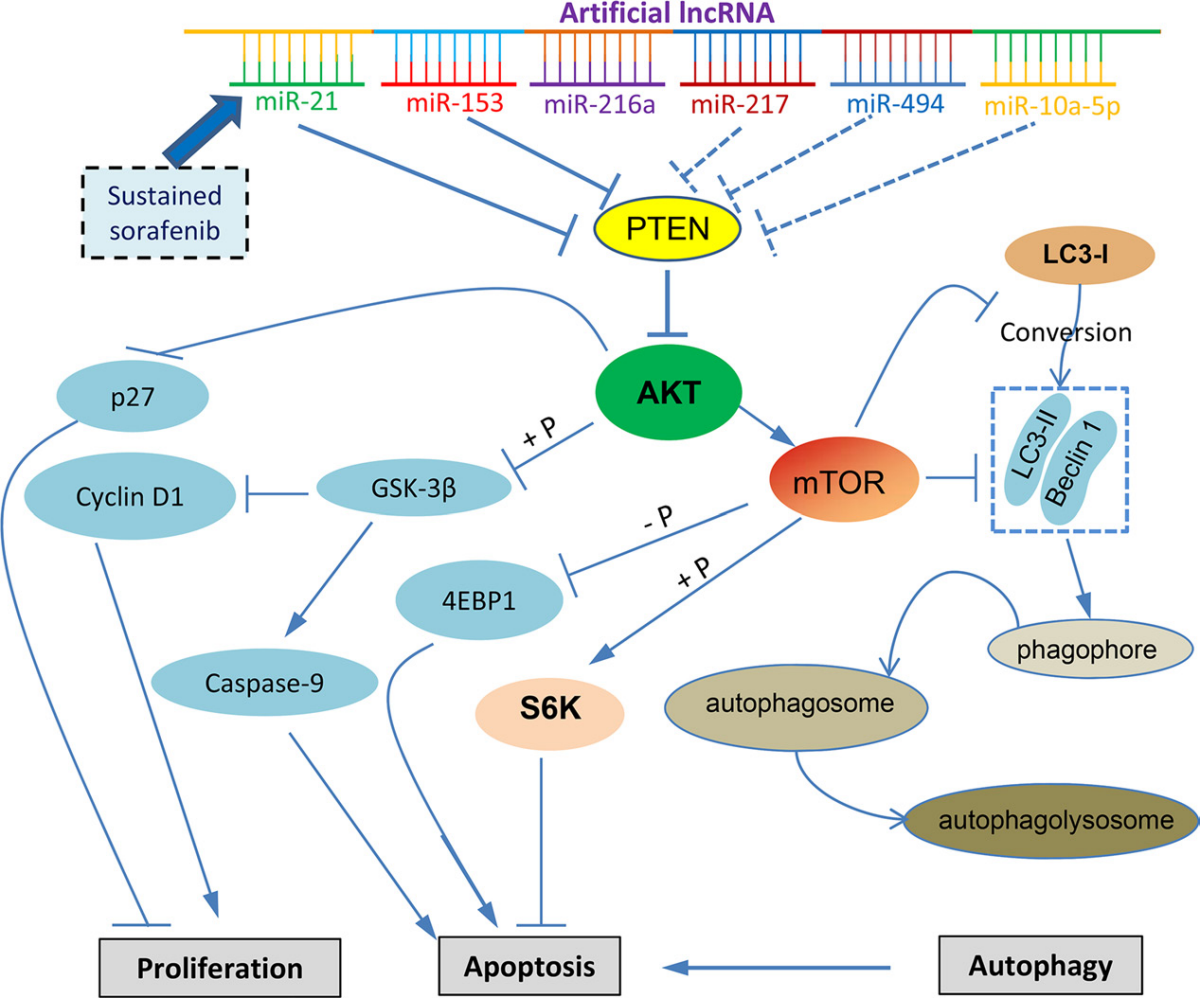
Proposed mechanisms by which an artificial lncRNA targeting multiple miRNAs to overcome the molecular mechanism contributing sorafenib resistance by regulating the PTEN/AKT pathway “→” indicates positive regulation or activation; “⊥”, negative regulation or blockade; “+ P”, regulation by phosphorylation. A dotted line indicates the mechanisms not investigated in the present study. Abbreviations: 4EBP1, eukaryotic translation initiation factor 4E-binding protein 1; GSK-3β, glycogen synthase kinase 3β; LC3, microtubule-associated protein 1 light chain 3; mTOR, mammalian target of rapamycin; PTEN, phosphatase and tensin homolog; S6K, ribosomal protein S6 kinase.

Sorafenib plays a critical role in the treatment of advanced HCC as it remains the unique systemic drug of choice [4]. Unfortunately, sorafenib has demonstrated low survival benefits, and some HCC patients initially respond to sorafenib but eventually succumb to the disease [23]. Therefore, it is imperative to investigate the putative mechanisms which underlie the acquired resistance to sorafenib to help develop potential strategies aimed at increasing its efficacy against HCC. In the present study, we have designed an artificial lncRNA, which simultaneously targets multiple miRNAs including miR-21, miR-153, miR-216a, miR-217, miR-494 and miR-10a-5p (Figure 7). These miRNAs participate in the molecular mechanisms of sorafenib resistance and targeting each has been shown to reverse drug resistance in HCC cells [8, 11–15, 19]. The AlncRNA expressed by an adenoviral vector, Ad5-AlncRNA, inhibited the regulatory functions of target miRNAs, and significantly induced growth inhibition and apoptosis of sorafenib-resistant cells and enhanced the effects of sorafenib *in vitro* and in animal models. The present results are supported by two recently published studies, in which an artificial lncRNA (Different names were given) was designed to target multiple oncomiRNAs, and shown to exert antitumor efficacy in treating HCC [24] or diffuse large B-cell lymphoma [25].

Overexpression and dysfunction of miRNAs have been well documented in carcinogenesis and progression of cancers by regulating oncogenes and suppressor oncogenes [26]. MiR-21 is one of the few widely studied miRNAs expressed in many types of cancer including HCC, and plays a central role in cancer progression [8, 27]. MiR-21 has also been identified as a key miRNA associated with drug resistance including the resistance to sorafenib [21]. MiR-153 [12], miR-216a/217 [13] and miR-494 [15] have been reported to contribute to sorafenib resistance in HCC cells. MiR-10a has been found to be overexpressed in lung cancer by directly targeting PTEN [20], and contributes to doxorubicin resistance in breast cancer cells [28]. The role of the above six miRNAs in the resistance of HCC cells to sorafenib has been further confirmed in the present study, and thus they have been selected as miRNA targets for designing the AlncRNA. MiR-222 has also been shown to contribute to sorafenib resistance of HCC cells through activating the PI3K/AKT signaling pathway [14]. However, the role of miR-222 has not been verified in this study, and was excluded in designing the AlncRNA.

We and others have previously demonstrated that AKT is highly activated in sorafenib-resistant HCC cells [8, 11, 12, 14, 15, 19]. PTEN is a well characterized tumor-suppressing phosphatase that inhibits AKT activation [29]. Previous studies have revealed that PTEN appears to be a common target for the 6 miRNAs noted above [8, 11–13, 15, 20, 30]. Here we have again shown that sorafenib induces downregulation of PTEN, and the activation of AKT in HCC cells. These studies have emphasized the importance of the PTEN/AKT pathway in the molecular mechanisms of sorafenib resistance.

Anti-miRNA oligonucleotides have been used to block the function of miRNAs. To confirm the efficiency in blocking miRNA functions by the AlncRNA, we used two luciferase reporters containing the 3′-UTR of PTEN with a miR-21 or miR-153 seed sites. AlncRNA was shown to be superior to either anti-miR-21 or miR-153 in inhibiting luciferase activities. The results indicate that AlncRNA simultaneously targets multiple miRNAs and block their function, and showed a stronger activity in regulating PTEN than each miRNA. MiRNAs bind to the mRNA of targeted genes to induce mRNA degradation or inhibit mRNA translation [6]. Here we have also demonstrated that miR-21 downregulates the expression of PTEN by preventing mRNA from being translated, while miR-153 could mediate PTEN mRNA degradation.

Autophagy was initially referred as a self-digestion process, but it is now also considered to be Type II programmed cell death under certain circumstances [31]. During the acquired drug resistance, autophagy switches from a protective function to a death-promoting role [21, 32, 33]. Activation of AKT induced by sorafenib exposure upregulates the expression of mTOR, which exerts an inhibitory effect on autophagy by dysregulating autopahgic proteins Beclin 1 and LC3 [33]. Here, we have shown that AlncRNA induces cell autophagy by targeting the PTEN/AKT pathway. Inhibition of autophagy counteracts the effects of AlncRNA, while induction of autophagy promotes cell autophagic death and augments its effects against sorafenib-resistant HCC cells.

One limitation of the present study is that the pattern of miRNA expression has not been validated in clinical sorafenib-resistant HCC tissues. According to the guideline of American Association for the Study of Liver Diseases (AASLD), sorafenib treatment is indicated for advanced HCC patients, who have lost the opportunity for curative therapies [34]. It appears almost impossible to collect sorafenib-resistant HCC tissues from these late-staged patients by laparotomy or needle biopsy as the patients cannot benefit from the procedures. Postmortem may be the only possible procedure for the collection of sorafenib-resistant HCC tissues.

In summary, we have designed an artificial lncRNA, which contains tandem antisense sequences specifically binding to the complementary sequences of miRNAs. The 6 target miRNAs contribute to the molecular mechanisms of sorafenib resistance in HCC cells by commonly dysregulating PTEN, and sequentially resulting in the activation of AKT (Figure 7). The proposed cellular signaling of AKT and its downstream factors, including apoptotic proteins, S6K, GSK3β and 4EBP1, and cell proliferation proteins, p27 and cyclin D1, have been well studied and documented (Figure 7) [35]. Activated AKT promotes the expression of mTOR, leading to the inhibition of the conversion of LC3-I to LC3-II, which is critical for cell autophagy [36]. The AlncRNA delivered by an adenoviral vector inhibits the activation of AKT by blocking the functions of the miRNAs, and enhances the efficacy of sorafenib in suppressing HCC cells. The results indicate that targeting multiple miRNAs by using an artificial lncRNA could be a promising strategy for overcoming sorafenib resistance in the treatment of HCC.

## MATERIALS AND METHODS

Please refer to Supplementary Materials for more detailed descriptions for Cell culture, antibodies and reagents, MiRNA Microarray and real-time PCR miRNA quantification, Polymerase chain reaction (PCR) analyses, Cell proliferation analysis, *In vitro* apoptosis assay, Immunoblotting analysis, Autophagy assays, Immunohistochemistry and *In situ* Ki-67 proliferation index, *In situ* detection of apoptotic cells.

### Establishment of sorafenib-resistant cells

The half maximal inhibitory concentration (IC_50_) of cells was determined by incubating cells with different concentrations of sorafenib, and cell viability was measured 3 days later. Cells were then cultured in 6-well plates at 1 × 10^4^ cells/well and incubated with sorafenib at a concentration just below their respective IC_50_. The concentration of sorafenib was slowly increased by 0.25 μM per week. After 6 months, two sorafenib-resistant cell lines, termed HepG2-SR and Huh7-SR, were generated from human HCC cell lines, HepG2 and Huh7, respectively. The sorafenib-resistant cells were continuously maintained by culturing them in the presence of sorafenib.

### Construction of AlncRNA expression vectors

A tandem sequence containing 6 copies of complementary sequences binding to the selected miRNAs (miR-10a-5p, miR-21, miR-153, miR-216a, miR-217 and miR-494) was synthesized and used as the encoding sequence for the AlncRNA. A CACC-box and *Eco*RI site were introduced at the 5′-end, and a *Bam*HI site introduced at the 3′-end. A stop codon (TAG) was introduced into the beginning and 3′ end of the AlncRNA sequence. The sequence was inserted into the *Eco*RI/*Bam*HI sites of plasmid pDC315 to construct an AlncRNA-expressing plasmid pDC315-AlncRNA. This plasmid and the adenovirus packaging plasmid pAd5F35 were co-transfected into HEK293 cells to recombine Ad5-AlncRNA. The previously constructed Ad5-EGFP armed with the enhanced green fluorescent protein (EGFP) was used as a negative control adenovirus [37]. The recombinant adenoviruses propagated in HEK293 cells were purified by cesium chloride density gradient centrifugation. The infectious viral titer was determined by measuring the median tissue culture infective dose (TCID50).

### Detection of adenovirus infection efficiency

Cells were seeded in 96-well plates (1 × 10^4^ cells/well) and cultured for 24 h. Then culture media were replaced with fresh media without FBS. Ad5-AlncRNA viruses were added at different multiplicities of infection (MOI), and Ad5-EGFP viruses served as controls. Three h later, cells were re-fed with media containing 5% FBS. Cells were harvested 0, 24, 48 and 72 h later, and the viral titer was measured with the TCID50 method. Parallel cells infected with Ad-EGFP viruses were observed under fluorescent microscopy.

### Luciferase reporter assay

To evaluate the function of miR-21 or miR-153, the 3′UTR of PTEN with a miR-21 or miR-153 targeting sequence was cloned into a pMIR-REPORT luciferase reporter vector (Ambion). The assay was conducted as described previously [11]. Briefly, the reporter vector plasmid was transfected into cells by using Lipofectamine 2000. To correct transfection efficiency, a luciferase reporter vector without the miRNA target was transfected in parallel. Cellular luciferase activities were measured by using a luciferase assay kit (Promega, Madison, WI), and the miR-21 or miR-153 function was expressed as percentage of the luciferase activity of the reporter vector with miRNA-targeting sequence over the one without the miRNA-targeting sequence.

### Transfection of oligonucleotides

Anti-miR-21 (5′-UCAACAUCAGUCUGAUAA GCUA-3′), anti-miR-153 (5′-GAUCACUUUUGUGA CUAUGCAA-3′), and negative control oligonucleotides were purchased from GenePharma Co., Ltd., Shanghai, China). Cells were grown to 60–70% confluence, and incubated with RNAs at a final concentration of 0.1 μM by using Lipofectamine™ 2000 (Invitrogen, Beijing, China) in a serum-free medium for 48 h and then subjected to assays.

### Animal experiments

Six to 8-week-old male nude BALB/c mice (H-2b) were obtained from the Animal Research Center, The First Affiliated Hospital of Harbin Medical University, China. This study had been approved (permit SYXK20020009) by the Animal Ethics Committee of Harbin Medical University, in compliance with the Experimental Animal Regulations by the National Science and Technology Commission, China. The experimental protocol has been described previously [11, 21]. Briefly, Huh7-SR cells (5 × 10^6^) were inoculated subcutaneously into the back of 45 mice, which received oral administration of 10 mg/kg sorafenib every three days. A lower dose of sorafenib was used in order to maintain the sorafenib-resistant ability of injected Huh7-SR cells, which were kept in the presence of sorafenib in culture. Thirty days later (when the tumors reached ~100 mm^3^), 38 mice (38 out of 45) were assigned to four treatment groups (7–8 mice per group), namely control, Ad5-EGFP, sorafenib, Ad5-AlncRNA and combination. Sorafenib was suspended in an oral vehicle containing Cremophor (Sigma-Aldrich), 95% ethanol and water in a ratio of 1:1:6 [11, 21, 38], and administrated orally at a dose of 30 mg/kg by daily gavage. Ad5-EGFP or Ad5-AlncRNA viruses were injected intratumorally at a dose of 2′ 10^8^ pfu/100 μl every other day for a total of five times. Mice in the control group received oral vehicle while mice in the combination group received oral sorafenib and intratumoral Ad5-AlncRNA viruses. Two mice from the control and Ad5-EGFP groups were sacrificed 10 days later and tumors harvested for analysis. The remaining mice were monitored for the tumor size once every 3 days and killed 21 days following commencement of treatment.

### Statistical analysis

All data are presented as the mean ± standard deviation. Comparisons of the paired data were carried out using *t-test*, and comparisons among multiple groups were carried out using one-way analysis of variance followed by Dunnet's *t-test* with the SPSS 13.0 software package. *P* < 0.05 indicates statistical significance.

## SUPPLEMENTARY MATERIALS TABLES AND FIGURES


