# Supplementary material for: An artificial lncRNA targeting multiple miRNAs overcomes sorafenib resistance in hepatocellular carcinoma cells
Source: Oncotarget. 2016 Sep 28;7(45):73257–69. doi: 10.18632/oncotarget.12304 (PMC5341977; doi:10.18632/oncotarget.12304)
Supplement: Supplementary file 1 [file oncotarget-07-73257-s001.pdf]

An artificial lncRNA targeting multiple miRNAs overcomes sorafenib resistance in hepatocellular carcinoma cells

Supplementary Materials

Cell culture, antibodies and reagents

Human HCC HepG2 cells were obtained from the American Type Culture Collection (ATCC, Manassas, VA, USA), and Huh7 cells from Chinese Academy of Sciences Cell Bank (Shanghai, China). Cells were cultured at 37°C in Dulbecco's modified Eagle's medium (DMEM) (Gibco BRL, Grand Island, NY, USA) supplemented with 10% fetal bovine serum (FBS). The antibodies (Abs) against AKT, p-AKT (Ser473), glycogen synthase kinase (GSK) 3β, phosphorylated GSK3β (p-GSK3β) (Ser9), mammalian target of rapamycin (mTOR), phosphorylated mTOR (p-mTOR) (Ser2448), ribosomal protein S6 kinase (S6K), phosphorylated S6K (p-S6K) (Thr389), eukaryotic translation initiation factor 4E-binding protein (4EBP)1, phosphorylated 4EBP1 (p-4EBP1) (Ser65), microtubule-associated protein 1 light chain 3 (LC3) and Beclin-1 were purchased from Cell Signaling Technology (Danvers, USA). The Abs against PTEN, $p27^{kip1}$, cyclin D1, caspase-3 and -9 and β-actin were from Santa Cruz Biotechnology (CA, USA). An anti-Ki67 Ab was from Abcam (Cambridge, MA, USA). Sorafenib was from Jinan Trio Pharmatech Co., Ltd. (Jinan, China). Rapamycin (RAP), 3-Methyladenine (3-MA), E-64d, pepstatin A and acridine orange were from Sigma-Aldrich (Shanghai, China). Sorafenib and RAP were dissolved in dimethyl sulfoxide (DMSO) to make a stock solution of 100 mM and 1 mM for *in vitro* assays, respectively. 3-MA was dissolved in PBS at a concentration of 200 mM by heating to 60–70°C immediately before use.

MiRNA microarray and real-time PCR miRNA quantification

Total RNA was extracted from cells by using Trizol reagents (Invitrogen). After reverse transcription with a TaqMan MicroRNA Reverse Transcription kit (Applied Biosystems), PCR array assays were performed by using TagMan Human MicroRNA Array A + B cards set v3.0 (Applied Biosystems). The 754 human mature miRNAs and 3 endogenous controls were reversely transcribed using Megaplex PreAmp primers. The reverse transcription products were subsequently loaded onto the TaqMan array to do real-time PCR amplification by using MX3000P Real-time PCR system (Stratagen, USA). The expression of miRNAs was measured by the ΔΔCt methods. The ΔCt was calculated by subtracting the Ct of U6 RNA from the Ct of each miRNA of interest. The ΔΔCt was calculated by subtracting the ΔCt of the control sample (parental cells) from the ΔCt of each sample (sorafenib-resistant cells). Fold change was generated by using the equation $2^{-\Delta\Delta Ct}$. To validate the expression levels of miRNAs measured by microarray, several selected miRNAs and U6 (an inner control) were further examined by real-time PCR by using a TaqMan MiRNA Reverse Transcription Kit (Applied Biosystems), individual TaqMan MiRNA assay, and MX3000P Real-time PCR system.

Polymerase chain reaction (PCR) analyses

Total RNA was extracted from cells. Conventional reverse transcription (RT)-PCR and quantitative real-time PCR (qRT-PCR) were both used to detect AlncRNA expression with a pair of primers (Forward: 5′-GACATCAGTCTGATAAGCTAA-3′) and (Reverse: 5′-GTTGTCCGTGTTGTTACCCTG-3′). For detecting PTEN mRNA expression, a pair of primers (Forward: 5′-CAAGATGATGTTTGAAACTATTCCAATG-3′ and Reverse: 5′-CCTTTAGCTGGCAGACCACAA-3′) was used. The expression of GAPDH mRNA (Forward: 5′-CACCCATGGCAAATTCCATGGCA-3′ and Reverse: 5′-TCTAGACGGCAGGTCAGGTCCACC-3′) was used as an internal control. The PCR products were analyzed by MX3000P Real-time PCR systems (Stratagen, USA), and data were calculated by ΔΔCt methods as above.

Cell proliferation analysis

Cells were seeded into a 96-well plate (3×10^3/well) in triplicate, and cultured overnight. The culture medium was replaced with fresh FBS-free media containing vehicle or test reagents at various concentrations over different times. Cell viability was measured with a Cell Counting Kit-8 (CCK-8) kit (Dojindo Molecular Technologies, Gaithersburg, MD, USA). Untreated cells served as controls. Cell viability (%) was calculated according to the formula: experimental OD value/control OD value ×100%.

In vitro apoptosis assay

Cells (1×10^5) were suspended in 100 µl binding buffer, 5 µl of Annexin V and 5 µl of propidium iodide (PI)

were added, and incubated for 15 min at room temperature in the dark, according to the manufacturer's instruction (BD Biosciences, San Jose, CA). Cells were subjected to flow cytometry to measure the rate of apoptosis (%) with a Beckman Coulter Epics Altra II cytometer (Beckman Coulter, California, USA).

Immunoblotting analysis

Cells or tissues were homogenized in protein lysate buffer (50 mM Tris pH 7.4, 100 µM EDTA, 0.25 mM sucrose, 1%SDS, 1% NP40, 1 µg/ml leupeptin, 1 µg/ml pepstatin A and 100 µM phenyl methyl sulfonyl flouride) and debris was removed by centrifugation at 10,000 × g for 10 min at 4°C. Protein concentrations were determined (Bio-Rad, Richmond, CA, USA). Lysates were resolved on sodium dodecyl sulfate-polyacrylamide (SDS-PAGE) gels, electrophoretically transferred to polyvinylidene difluoride (PVDF) membranes. The membranes were blocked in TBST (137 mM NaCl, 20 mM Tris HCl [pH 7.6], and 0.1% [v/v] Tween 20) containing 5% (w/v) nonfat dry milk at 37°C for 2 h, and then incubated overnight with primary Abs, and subsequently with alkaline phosphatase-conjugated secondary Abs for 2 h at room temperature in the dark. They were developed with 5-bromo-4-chloro-3-indolyl phosphate (BCIP)/ nitro blue tetrazolium (NBT) (Tiangen Biotech Co. Ltd., Beijing, China). The density of each band was measured using a densitometric analysis program (FR200, Shanghai, China), and normalized to that of β-actin from the same cells. In preliminary experiments, serial dilutions of lysates (containing 2.5, 5, 10, 20, 40 or 80µg protein) were immunoblotted; band intensities were measured and plotted against protein amounts to generate a standard curve, and the amount of protein for each blot was determined.

Autophagy assays

Cells were incubated with acridine orange (5 µM) at 37°C for 15 min, washed with cold PBS, and examined by fluorescent microscopy. Acidic vesicular organelles (AVOs) appeared as orange/red fluorescent cytoplasmic vesicles, while nuclei were stained green. Acridine orange-stained cells were further trypsinized and analyzed on a FACScalibur flow cytometer (BD. Biosciences, San Jose, California, USA). The degree of autophagic lysosome was expressed as fold change of acridine orange fluorescence intensity (FL3) of red in treated cells versus control cells.

Immunohistochemistry and *In situ* Ki-67 proliferation index

Formalin fixed tumor specimens were transferred to 70% ethanol, and subsequently paraffin-embedded and sectioned. Tumor sections were rinsed with PBS, blocked with 3% BSA for 2 h, and incubated with primary Abs at 4°C overnight. They were subsequently incubated for 30 min with appropriate secondary Abs using the Ultra-Sensitive TMS-P kit (Zhongshan Co., Beijing, China), and immunoreactivity developed with Sigma FAST DAB (3,3′-diaminobenzidine tetrahydrochloride) and $CoCl_2$ enhancer tablets (Sigma-Aldrich, Shanghai, China). Sections were counterstained with hematoxylin, mounted, and examined by microscopy. The Ki-67 positive cells were counted microscopically in 10 randomly selected at 400 × objective magnification. The Ki-67 proliferation index was calculated according to the following formula: the number of Ki-67 positive cells/ the total cell count ×100%.

In situ detection of apoptotic cells

The above tumor sections were stained with the TUNEL (Terminal deoxynucleotidyl transferase dUTP nick end labelling) (Roche, Shanghai, China). TUNEL positive cells were counted by microscopy in 20 randomly selected fields at 200 × objective magnification. The apoptosis index was calculated according to the following formula: the number of apoptotic cells × /total number of nucleated cells × 100%.

Supplementary Table S1: Upregulation of miRNAs in sorafenib-resistant vs. parental HCC cells

miRNAs	Fold change (HepG2-SR vs. HepG2)	*P*-Value	Fold change (Huh7-SR vs. Huh7)	*P*-Value
miR-21*	9.351	< 0.001	12.234	< 0.001
miR-153*	6.512	0.003	8.945	< 0.001
miR-216a*	4.785	0.001	6.647	0.002
miR-217*	5.252	0.004	4.127	0.015
miR-494*	7.012	< 0.001	5.031	0.005
miR-10a-5p*	3.143	0.019	2.328	0.048
miR-1291	2.148	0.069	3.542	0.034
miR-223	2.463	0.043	3.025	0.069
miR-10b-5p	3.012	0.019	2.172	0.058
miR-195	2.837	0.029	1.451	0.123
miR-664	2.604	0.051	2.368	0.044
miR-34-a	3.022	0.042	2.135	0.063
miR-1260	1.278	0.096	2.465	0.028
miR-30a-3p	2.015	0.039	1.453	0.163
Let-7c	1.253	0.139	0.406	0.047
miR-1274a	1.729	0.517	1.032	0.141
miR-219-1-3p	1.933	0.328	0.582	0.055
Let-7b	0.463	0.033	1.069	0.965
miR-222	0.621	0.527	0.352	0.036
miR-616	0.380	0.059	0.256	0.042

Note: Sorafenib-resistant and parental HCC cells (HepG2-SR and HepG2, Huh7-SR and Huh7) were subjected to real-time PCR. The expression level of each miRNA was measured, and fold change was calculated. P value < 0.05 was considered statistically significant. "*" indicates a significant increase in miRNA expression between sorafenib-resistant vs. parental cells in both pairs of HCC cells.

Supplementary Table S2: Six targeted miRNAs and their complementary binding sequence

MiRNA	Targeting genes	Sequence	Complementary binding sequence in the AlncRNA
miR-21	PTEN, RECK, TIMP3, PDCD4 & TPM1 [1, 2]	5′-UAGCUUAUCAGACUGAUGUUGA-3′	5′-ACATCAGTCTGATAAGCTA-3′
miR-153	PTEN, FOXO1, Survivin & Bcl-2 [3]	5′-UUGCAUAGUCACAAAAGUGAUC-3′	5′-ACTTTTGTGACTATGCA-3′
miR-216a	PTEN & SMAD7[4]	5-UAAUCUCAGCUGGCAACUGUGA-3′	5′-ACAGTTGCCAGCTGAGA-3′
miR-217	PTEN & SMAD7 [4]	3′-UACUGCAUCAGGAACUGAUUGGA-5′	5′-ATCAGTTCCTGATGCAG-3′
miR-494	PTEN [5]	5′-AGGUUGUCCGUGUUGUCUUCUCU-3′	5′-GACAACACGGACAACCT-3′
miR-10a-5p	PTEN [6]	5′-UACCCUGUAGAUCCGAAUUUGUG-3′	5′-CGGATCTACAGGGTA-3′

Notes: FOXO1, forkhead box protein O1; PDCD4, programmed cell death 4; PTEN, phosphatase and tensin homolog; RECK, reversion.
Inducing cysteine rich protein with kazal motifs; SMAD7, mothers against decapentaplegic homolog 7; TIMP3, TIMP metallopeptidase.
inhibitor 3; TPM1, tropomyosin 1.

REFERENCES

1. He C, Dong X, Zhai B, Jiang X, Dong D, Li B, et al. MiR-21 mediates sorafenib resistance of hepatocellular carcinoma cells by inhibiting autophagy via the PTEN/Akt pathway. Oncotarget. 2015; 6:28867–81.
2. Meng F, Henson R, Wehbe-Janek H, Ghoshal K, Jacob ST, Patel T. MicroRNA-21 regulates expression of the PTEN tumor suppressor gene in human hepatocellular cancer. Gastroenterology. 2007; 133:647–58.
3. Chen Y, Feng F, Gao X, Wang C, Sun H, Zhang C, et al. MiRNA153 Reduces Effects of Chemotherapeutic Agents or Small Molecular Kinase Inhibitor in HCC Cells. Current cancer drug targets. 2015; 15:176–87.
4. Xia H, Ooi LL, Hui KM. MicroRNA-216a/217-induced epithelial-mesenchymal transition targets PTEN and SMAD7 to promote drug resistance and recurrence of liver cancer. Hepatology (Baltimore, Md). 2013; 58:629–41.
5. Liu K, Liu S, Zhang W, Jia B, Tan L, Jin Z, et al. miR-494 promotes cell proliferation, migration and invasion, and increased sorafenib resistance in hepatocellular carcinoma by targeting PTEN. Oncology reports. 2015; 34:1003–10.
6. Yu T, Liu L, Li J, Yan M, Lin H, Liu Y, et al. MiRNA-10a is upregulated in NSCLC and may promote cancer by targeting PTEN. Oncotarget. 2015; 6:30239–50.

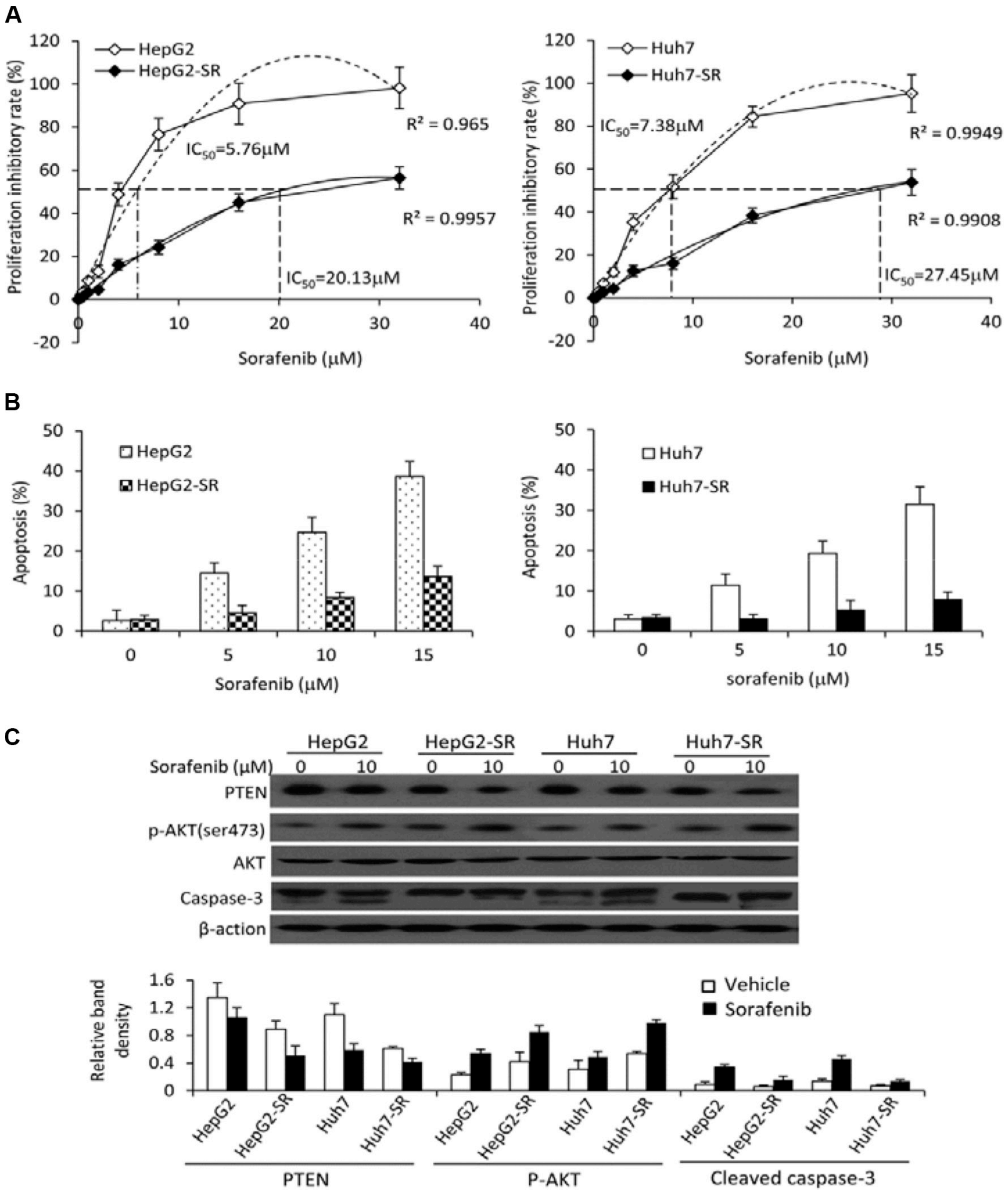

Supplementary Figure S1: Sorafenib-resistant HCC cells are insensitive to sorafenib by activating the PTEN/Akt pathway. (**A**) HepG2, HepG2-SR, Huh7 and Huh7-SR cells were incubated with serial concentrations of sorafenib for 48 h. Untreated cells served as controls. The viability of cells was assessed to calculate the proliferation inhibitory rate (%). The linear regression was performed and the R^2 value was calculated. The dotted curves show the trend of dose-dependent cell proliferation inhibition rates, and the dotted lines indicate drug concentrations, which resulted in 50% of maximal proliferation inhibition (IC_{50}) of cells. (**B**) The above cells were incubated with sorafenib (0, 5, 10 or 15 μM) for 48 h, and then analyzed cytometrically to detect apoptosis. (**C**) Lysates from the above cells incubated with sorafenib (0, 10 μM) were immunoblotted. The density of each band was measured and normalized to respective β-actin.

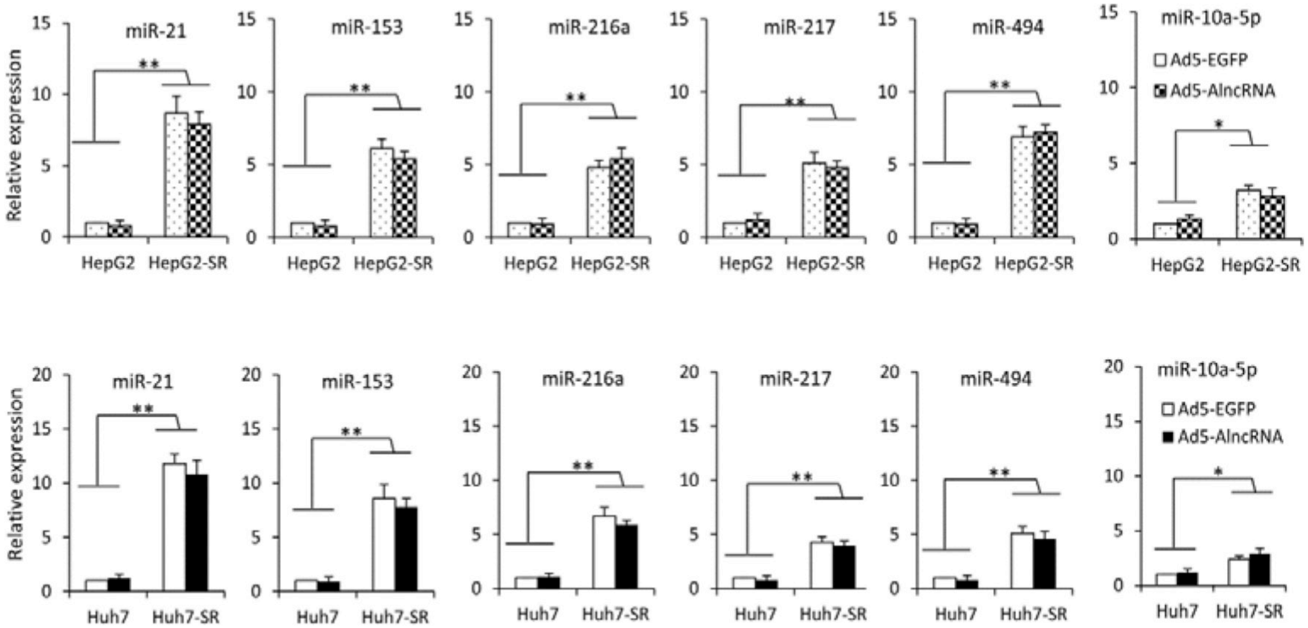

Supplementary Figure S2: Infection of Ad5-AlncRNA has little effect on the expression of targeted miRNAs. HepG2, HepG2-SR, Huh7 and Huh7-SR cells were infected with Ad5-AlncRNA at an MOI of 100 pfu/cell for 3 h. Cells were harvested and subjected real-time PCR to quantify the expression of microRNAs as indicated. "*" ($P < 0.05$) and "**" ($P < 0.001$) indicate a significant difference.

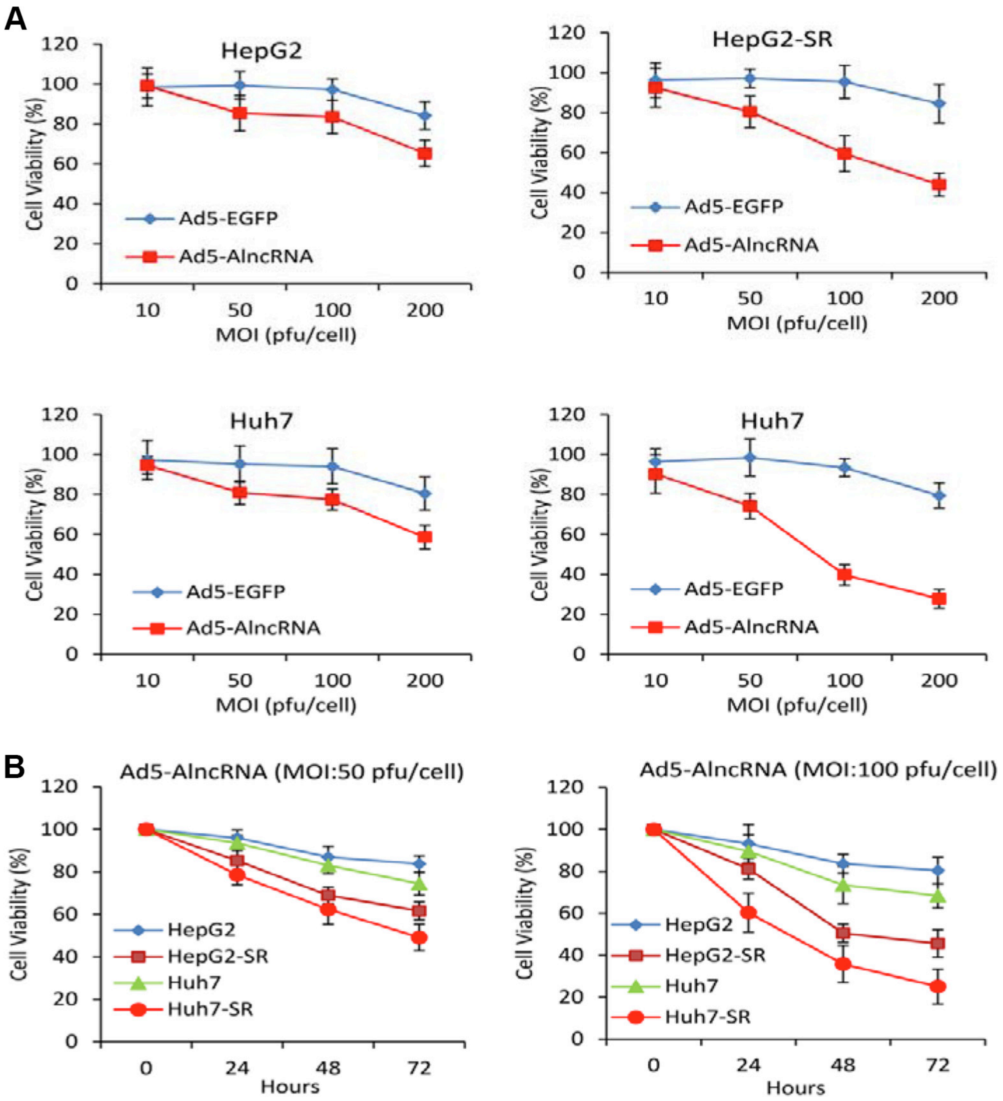

Supplementary Figure S3: Infection of Ad5-AlncRNA showed a stronger inhibitory effect on the growth of sorafenib-resistant cells in dose- and time-dependent manners. (**A**) HepG2, HepG2-SR, Huh7 and Huh7-SR cells were seeded in 96-well plates at 1×10^3 cells/well, and incubated for 48 h with Ad5-AlncRNA or Ad5-EGFP at MOIs of 10, 50, 100 or 200 pfu/cell. Cell viability (%) was measured and normalized with the corresponding untreated cells. (**B**) The above cells were incubated with Ad5-AlncRNA at an MOI of 50 or 100 pfu/cell for 24, 48 or 72 h. Cell viability (%) was measured and normalized with the corresponding cells at 0 h.

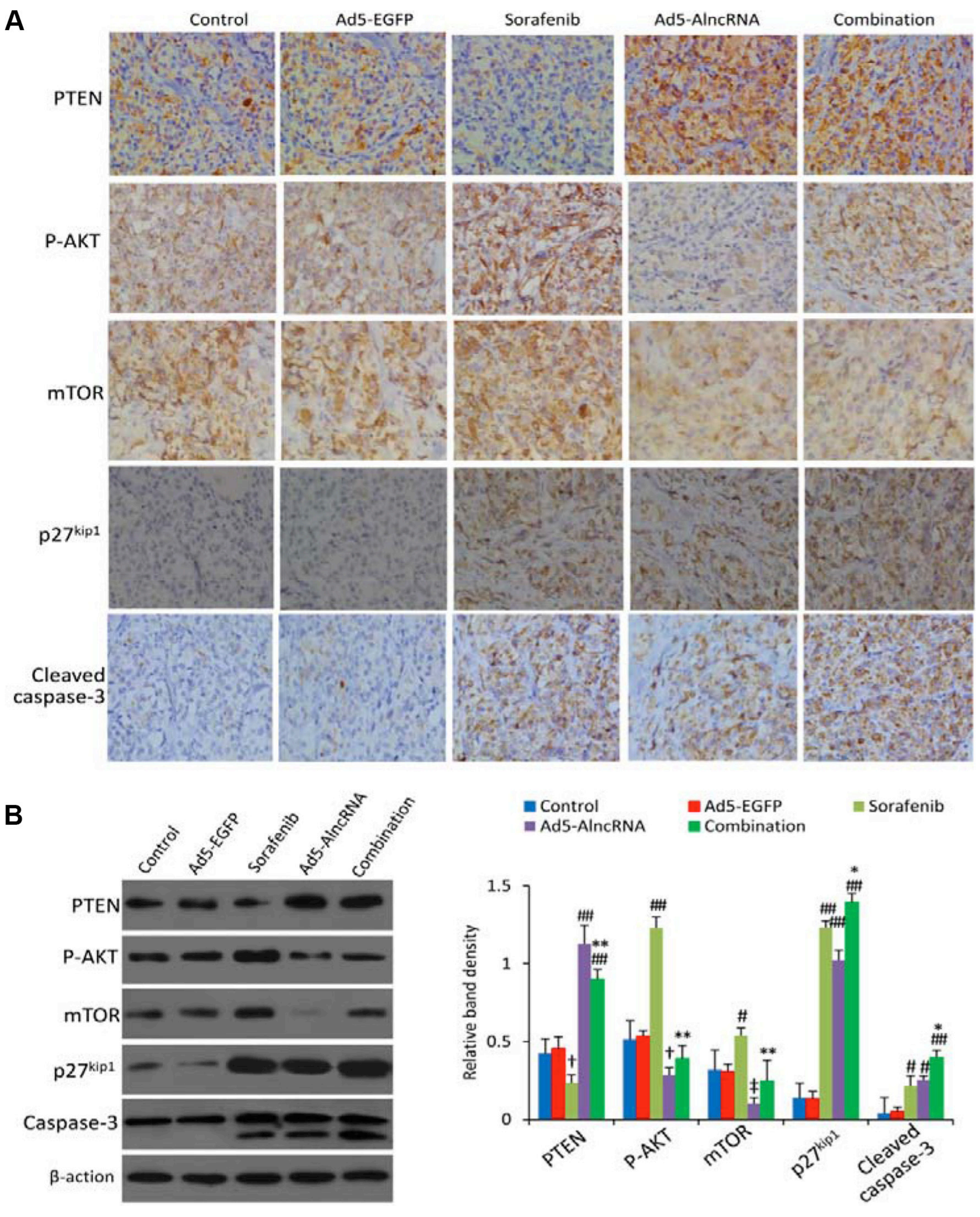

Supplementary Figure S4: Tumoral expression of target genes by miRNAs *in situ*. (**A**) The formalin-fixed, paraffin-embedded tumor sections harvested from Figure 6 were analyzed by immunohistochemistry for detecting the expression of miRNA targeted genes. (**B**) Tumor lysates were subjected to immunoblotting. The density of each band was measured and normalized to respective β-actin. "*" ($P < 0.05$) and "**" ($P < 0.001$) indicates a significant difference from sorafenib-treated tumors. "†" ($P < 0.05$) and "‡" ($P < 0.001$) indicate a significant reduction, while "#" ($P < 0.05$) and "##" ($P < 0.001$), a significant reduction, versus control tumors.
